# The effect of adding chewing gum to oral carbohydrates on preoperative anxiety scores in women undergoing gynecological surgery: A randomized controlled study

**DOI:** 10.1371/journal.pone.0283780

**Published:** 2023-04-25

**Authors:** Yu Jeong Bang, Jong-Hwan Lee, Chung Su Kim, Dan-Cheong Choi, Joseph J. Noh, Yoo-Young Lee, Jeong-Jin Min

**Affiliations:** 1 Department of Anesthesiology and Pain Medicine, Samsung Medical Center, Sungkyunkwan University School of Medicine, Seoul, Korea; 2 Department of Obstetrics and Gynecology, Division of Gynecologic Oncology, Samsung Medical Center, Sungkyunkwan University School of Medicine, Seoul, Korea; Aristotle University of Thessaloniki School of Veterinary Medicine, GREECE

## Abstract

**Background:**

Preoperative anxiety is an unpleasant experience that can adversely affect perioperative outcomes. Although clinical benefits of preoperative oral carbohydrate is well reported, the effect of adding chewing gum to carbohydrate loading has never been studied. We aimed to investigate the effect of adding gum-chewing to oral carbohydrates on preoperative anxiety and gastric volume in patients undergoing gynecologic surgery.

**Methods:**

One hundred and four patients were enrolled and randomized either into a carbohydrate drink group (CHD group) or CHD with gum group. The CHD group was instructed to drink 400 mL of oral carbohydrate the evening before and 200–400 mL 3 hours before surgery. The CHD with gum group was encouraged to chew gum freely during preanesthetic fasting in addition to consuming oral carbohydrates in the same manner. The primary endpoint was preoperative anxiety assessed using the Amsterdam preoperative anxiety and information scale (APAIS). The degree of patient-reported quality of recovery after surgery and gastric volume prior to general anesthesia were also compared as secondary outcomes.

**Results:**

Preoperative APAIS was lower in the CHD with gum group compared with the CHD group (16 [11.5, 20] vs. 20 [16.5, 23], p = 0.008). Patient-rated quality of recovery after surgery was also higher in the CHD with gum group and showed a significant negative correlation with preoperative APAIS score (correlation coefficient: -0.950, p = 0.001). Gastric volume were not different between the groups (0 [0–0.45] vs. 0 [0–0.22], p = 0.158).

**Conclusion:**

The addition of gum chewing to oral carbohydrate loading during preoperative fasting was more effective in relieving preoperative anxiety than oral carbohydrate alone in women patients undergoing elective gynecologic surgery.

**Trial registration:**

Clinical Research Information Services, CRIS identifier: KCT0005714, https://cris.nih.go.kr/cris/index.jsp.

## Introduction

Women undergoing gynecologic surgery tend to present with high levels of preoperative anxiety [1,2], thus an anxiety-reducing strategy is needed for this patient population. However, an effective method for preoperative anxiety relief has not yet been established [3–6]. Chewing gum has been found to improve depressive mood and reduce stress and anxiety [7–9]. Moreover, previous studies [10–12] confirmed that chewing gum during preanesthetic fasting neither affected gastric contents nor increased pulmonary aspiration risk. Therefore, the fasting guidelines from the European Society of Anesthesiology state that surgery should not be canceled or delayed because of gum chewing [13]. Furthermore, some opinions have recently emerged to allow or recommend chewing gum during the preoperative fasting period due to the potential benefits of alleviating anxiety and dry mouth [11,14,15].

According to the Enhanced Recovery After Surgery (ERAS) concept, preoperative oral carbohydrate loading is advantageous for perioperative metabolic optimization and reduces thirst, hunger, and anxiety [16]. However, to the best of our knowledge, the benefit of adding chewing gum to carbohydrate loading for preoperative anxiety relief has never been studied. We hypothesized that allowing chewing gum during preoperative fasting would further alleviate anxiety and patient discomfort without affecting the risk of pulmonary aspiration.

We aimed to evaluate the usefulness of chewing gum on preoperative anxiety relief and patient-reported outcomes during hospitalization in patients undergoing gynecologic surgery with the ERAS fasting protocol, including oral carbohydrate loading.

## Methods

### Study design

This study is a single-center, randomized, controlled study. The trial was performed at a tertiary medical center in Seoul, Korea between January 2021 and August 2021. Ethical approval for this study (SMC 2020-10-058-002) was provided by the Samsung Medical Center Institutional Review Board on December 3, 2020. The study was registered with the Clinical Research Information Services (CRIS identifier: KCT0005714) prior to patient enrollment on December 29, 2020. The study was conducted in accordance with the principles of the Declaration of Helsinki and the International Conference on Harmonization of Good Clinical Practice guidelines. Written informed consent was obtained from each participant and/or their legal guardian prior to inclusion.

### Study population

The patients undergoing elective gynecologic surgery were admitted the day before surgery, and invited to participate in the study protocol. We enrolled adult patients between 18 and 70 years old with American Society of Anesthesiologists Physical Status classification I to Ⅲ who were scheduled for elective laparoscopic gynecologic surgery. We excluded patients with emergent conditions, body mass index (BMI) > 30, previous gastrointestinal surgery, gastrointestinal disorder (e.g., gastroesophageal reflux, diabetic gastroparesis, ileus), or current medication affecting gastrointestinal motility [17–19].

### Randomization and blinding

Eligible patients were randomly assigned to either a carbohydrate drink group (CHD group) or a carbohydrate drink with chewing gum group (CHD with gum group) at a ratio of 1:1. Randomization was performed using a random permuted block design with a block size of two generated by the computer. Allocation information was sealed in an opaque envelope numbered with a randomization sequence and stacked in the education room of the gynecologic cancer center. An investigator (YJB) enrolled the patient and assessed the baseline anxiety. After obtaining informed consent, a gynecologist (YYL) opened the sealed envelope and distributed the instruction for gum chewing. The blinded investigator (YJB) performed an ultrasound scan for gastric antrum before induction. An independent anesthesiologist performed induction and supervised general anesthesia. The blinded researchers (YJB, MJJ) interviewed the patients about preoperative anxiety in the preoperative holding area, perioperative outcomes, and postoperative recovery during the in-hospital stay.

### Preoperative fasting

All participants were asked to follow the institutional fasting protocol based on the recommendations of the ERAS Society. The participants had to fast overnight before surgery, and oral carbohydrate drink (NO NPO^Ⓡ^: Daesang, Seoul, South Korea) loading was used routinely. All patients were encouraged to consume at least 600 mL to 800 mL of carbohydrate drinks up until 3 hours before induction of anesthesia. They received 400 mL of a carbohydrate drink (CHD) the evening before surgery and 200 mL of a carbohydrate drink at 5 am on the day of surgery or 3h before the anticipated anesthesia induction. The total amount and ingestion time of carbohydrate drinks were checked by an independent nursing team. Patients in the CHD group were requested to follow the aforementioned fasting guidelines without further treatment. Patients in the CHD with gum group were allowed to chew gum freely during the fasting period, following the fasting protocols described above. Participants in the CHD with gum group were provided with 24 pieces of sugarless gum (Xylichew^Ⓡ^: Xylichew, Hayden, ID, USA). Participants were instructed to chew gum freely until their departure for the operating room. It was encouraged for the patients to chew at least one piece of gum per hour except for when sleeping. However, participants could stop or take a break if symptoms such as jaw pain, toothache, or headache occurred. All participants in the CHD with gum group were asked to log their chewing time.

### Anesthesia and monitoring

Anesthetic providers were blinded to group allocation and used a standardized anesthetic protocol. No premedication was given to any of the participants. Anesthesia was induced with total intravenous (IV) anesthesia with propofol, remifentanil, and rocuronium (0.8 mg/kg) using target-controlled infusion (Orchestra® Base Primea; Fresenius Kabi, Brezins, France). The effect site concentration of propofol and remifentanil were adjusted to achieve a bispectral index of 40–60 and to maintain mean arterial blood pressure and heart rate to the pre-induction value. At the end of the surgery, neuromuscular blockade was antagonized with 250 μg/kg pyridostigmine and 10 μg/kg glycopyrrolate. All patients received IV patient-controlled analgesia (PCA) with fentanyl programmed to deliver 15 μg/h (1mL/h) with a 15-μg bolus (1mL) dose and a 15-minute lockout time.

### Postoperative management

In the surgical ward, all participants were managed according to a standardized protocol by independent gynecologists. All patients were encouraged to begin ambulation and chew gum freely after they completely recovered from anesthesia. Diet was resumed in the following order depending on what the patient could tolerate: sips of water, a soft diet, and then a normal diet. Pain severity was measured using a numeric rating scale (NRS; 0 = no pain, 10 = worst imaginable pain). IV PCA was continued until NRS ≤ 3. Despite IV PCA administration, if patients reported breakthrough pain (NRS > 5), 30 mg ketorolac or 1 g acetaminophen was administered in sequence. If this proved ineffective, 50mg of meperidine was administered intravenously. Postoperative nausea and vomiting (PONV) was treated with 10 mg metoclopramide and 0.3 mg ramosetron. Discharge criteria included oral intake of solid food and adequate pain control without the need for IV analgesics.

### Gastric fluid analysis

To rule out the risk of pulmonary aspiration due to chewing gum intervention, the estimated volume and acidity of gastric fluid were compared between the two groups. Before induction of anesthesia, a gastric ultrasound scan was performed on the operating table by a single experienced investigator (YJ Bang) to estimate gastric fluid volume. We measured gastric antrum cross-sectional area (CSA) using the free tracing method on the portable ultrasound unit. Gastric fluid volume was estimated by the following formula: Gastric fluid volume (mL) = 27.0 + 14.6 x Right–Lateral CSA (cm^2^)– 1.28 x age (yr) [20]. We collected gastric fluid drained naturally through the multi-orificed silicone thermometer, which was inserted for monitoring of core temperature and decompression of the stomach. Gastric fluid acidity was analyzed with a pH meter.

### Assessment of preoperative anxiety and self-reported outcomes

Our primary outcome was preoperative anxiety level evaluated by total Amsterdam preoperative anxiety and information scale (APAIS) score in the preoperative holding area on the operation day. The APAIS [21,22] consists of six items in two domains: the anxiety scale (4–20) and the need for information scale (2–10). Four items in the anxiety scale represent fear of anesthesia and the surgical procedure. Two items in the need for information scale represent coping styles for anxious situations. Higher APAIS scores indicate higher levels of preoperative anxiety. All patients rated their preoperative APAIS score twice, first in the ward a day before surgery for baseline anxiety and second in the preoperative holding area after fasting. The discomfort associated with anxiety was evaluated with eight items using an NRS: unpleasantness, hunger, thirst, mouth dryness, fatigue, headache, nausea, and lack of concentration (0 = no discomfort, 10 = worst imaginable discomfort). Secondary outcomes included PONV, opioid consumption, and any bowel complications during the hospital stay. Other parameters associated with postoperative recovery such as bowel function recovery (time to flatus), Quality of Recovery-15 questionnaire (QoR-15K) scores, and length of hospital stay were also collected. All patients completed a Korean version of the QoR-15K [23] 24 hours after surgery to assess the quality of recovery after surgery and anesthesia. QoR-15K scores range from 0 to 150, with higher scores representing better recovery. The investigator who assessed patients during the perioperative period was blinded to group allocation.

### Sample size calculation and statistical analysis

The sample size was calculated based on pilot study data. The mean APAIS score immediately before surgery was 20.5 and the standard deviation was 6.0 in patients undergoing gynecologic surgery with the ERAS protocol. We assumed that the APAIS score of the CHD with gum group compared to the CHD group would need to decrease by 20% for clinically meaningful significance to be detected. With an alpha of 0.05 and a beta of 0.9, 45 participants are required in each group using the independent two-sample t-test. We enrolled 52 participants in each group to account for an expected 15% attrition rate, with a total of 104. All continuous data were tested for normality by the Shapiro–Wilk test. Normally distributed data were presented as means (standard deviations [SD]) and analyzed using independent two-sample t-test. Skewed data were presented as median (interquartile range [IQR]) and analyzed using Wilcoxon’s rank sum test. For categorical variables, all data were summarized as the number (%) and differences between groups were analyzed using the chi-square test or Fisher’s exact test as appropriate. The association between QoR-15K and APAIS scores measured in the preoperative holding area was analyzed using a linear regression model. A value of p < 0.05 was considered statistically significant. The individual p-value of multiple comparisons was adjusted using Bonferroni post-correction. Statistical analysis was performed using SAS version 9.4 (SAS Institute Inc, Cary, NC, USA).

## Results

**Fig 1:**
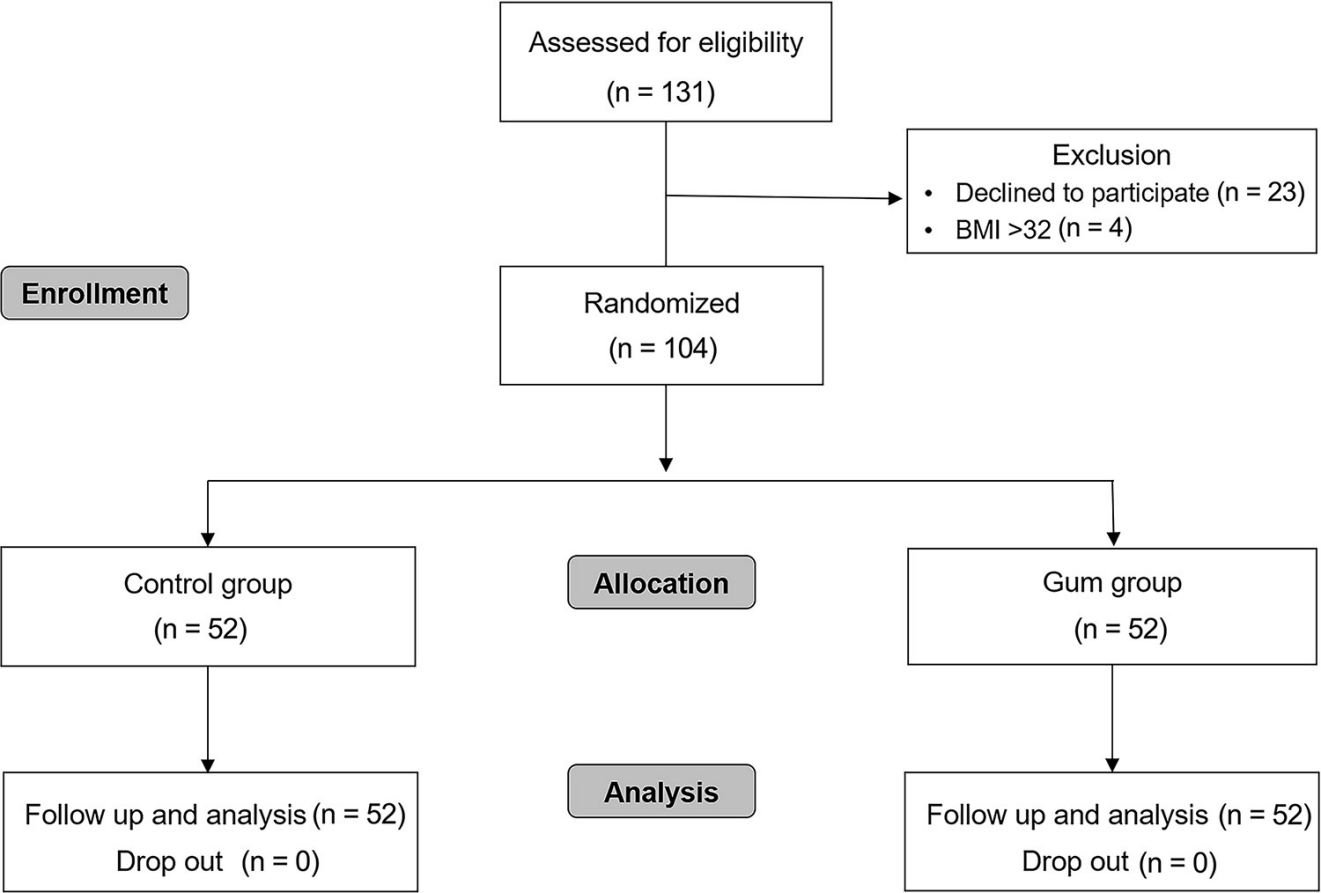
CONSORT flow diagram of patients included in the study. The control group comprised patients who followed preoperative fasting guidelines of the ASA and ERAS Society. The gum group comprised patients who were allowed to chew gum freely during the preoperative fasting period. ASA, American Society of Anesthesiologists; CONSORT, Consolidated Standards of Reporting Trials. ERAS, Enhanced Recovery After Surgery.

From January 2021 to August 2021, we screened 108 patients for eligibility. Of these, 4 were excluded due to obesity (BMI > 30). A total of 104 patients were enrolled and randomly assigned to the CHD group or CHD with gum group, and analyzed (Fig 1).

Baseline patient characteristics, including factors that could affect preoperative anxiety such as age, occupation, marital status, and previous experience with anesthesia or surgery were not different between the two groups (Table 1).

**Table 1:** Basic patient characteristics.

Parameter	Control group (N = 52)	Gum group (N = 52)
Age (yr), mean (SD)	44.6 (10.3)	44.6 (10.7)
Height (cm), mean (SD)	160.4 (4.9)	159.9 (5.0)
Weight (kg), median [IQR]	58.3 [52.2–63.7]	58.4 [53.7–65.4]
Body mass index (kg·m^-2^), median [IQR]	22.3 [20.4–25.1]	23.4 [20.6–25.7]
ASA physical status (I:II;Ⅲ), n (%)	30 (57.7%); 20 (38.5%); 2 (3.9%)	31 (59.6%); 18 (34.6%); 3 (5.8%)
Malignancy, n (%)	26 (50.0%)	26 (50.0%)
Baseline Anxiety (APAIS score), mean (SD)	16.7 (5.8)	15.5 (4.6)
Morbidity		
Hypertension	3 (5.8%)	6 (11.5%)
Diabetes mellitus	1 (1.9%)	1 (1.9%)
Chronic kidney disease	1 (1.9%)	1 (1.9%)
Liver disease	3 (5.8%)	4 (7.7%)
Current smoker	3 (5.8%)	1 (1.9%)
Occupation, n (%)		
Household duties	24 (46.2%)	20 (38.5%)
Clerical worker	11 (21.2%)	12 (23.1%)
Service worker	3 (5.8%)	5 (9.6%)
Professionals	7 (13.5%)	9 (17.3%)
Student	2 (3.8%)	1 (1.9%)
Retired or unemployed	5 (9.6%)	5 (9.6%)
Marital status, n (%)		
Married	12 (23.1%)	14 (26.9%)
Unmarried, divorced, or widowed	40 (76.9%)	38 (73.1%)
History of previous surgery, n (%)		
0; 1–2; ≥ 3	23 (44.2%); 24 (46.2%); 5 (9.6%)	25 (48.1%); 22 (42.3%); 5 (9.6%)
History of previous anesthesia, n (%)		
None	23 (44.2%)	25 (48.1%)
General anesthesia	28 (53.8%)	23 (44.2%)
Regional anesthesia	1 (1.9%)	4 (7.7%)

Abbreviations: APAIS, the Amsterdam preoperative anxiety and information scale (6 to 30); ASA, American Society of Anesthesiologists; IQR, interquartile range; SD, standard deviation.

### Preoperative anxiety and discomfort

**Fig 2:**
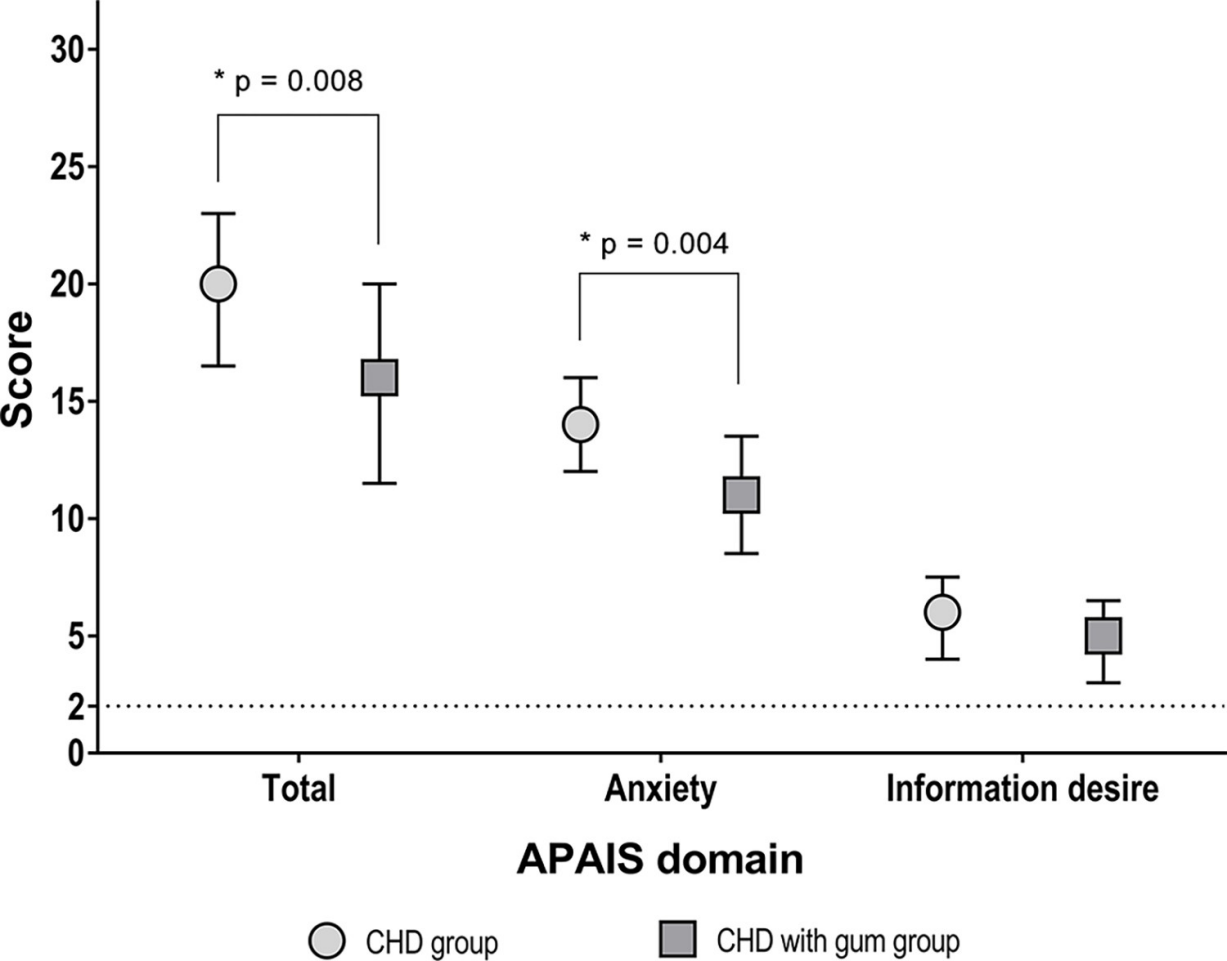
Comparison of preoperative anxiety score using the Amsterdam Preoperative Anxiety and Information Scale (APAIS). Data were analyzed using Wilcoxon’s rank sum test.

Preoperative anxiety was evaluated using the APAIS score (Table 2). The median total APAIS score was lower in the CHD with gum group compared to the CHD group (16 [11.5, 20] vs. 20 [16.5, 23], p = 0.008). The anxiety domain score of APAIS was also lower in the CHD with gum group than the CHD group (11 vs. 14, p = 0.004; Fig 2 and Table 2). Among items of preoperative discomfort (unpleasantness, hunger, thirst, mouth dryness, fatigue, headache, nausea, lack of concentration), the degree of hunger and unpleasantness in the control group were higher than those of the CHD with gum group (Table 2).

**Table 2:** Patient-reported outcomes.

	Control group (N = 52)	Gum group (N = 52)	p-value
APAIS—total, median [IQR]	20 [16.5–23]	16 [11.5–20]	0.008*
APAIS–anxiety, median [IQR]	14 [12–16]	11 [8.5–13.5]	0.004*
APAIS—information desire, median [IQR]	6 [4–7.5]	5 [3–6.5]	0.114*
Preoperative discomfort, median [IQR]			
Hunger	3 [0.5–6.5]	1.5 [0–4]	0.025
Thirst	3 [1–5	3 [1–5]	0.541
Mouth dryness	4 [2–5.5]	2 [1–5]	0.125
Fatigue	3 [0–5]	2 [0–5]	0.708
Headache	1 [0–3.5]	0.5 [0–2]	0.641
Nausea	0 [0–2]	0 [0–1]	0.142
Unpleasantness	2 [0–4.5]	1 [0–2]	0.022
Lack of concentration	3 [1–5]	2 [1–4]	0.088
Postoperative recovery, median [IQR]	111 [101.5–123.5]	130 [119–137.5]	< .0001*

Amsterdam preoperative anxiety and information scale (APAIS) indicates preoperative anxiety, 6–30.

Patients’ discomforts related to preoperative fasting and anxiety were rated using a numeric rating scale (NRS), 0 to 10.

Postoperative recovery was evaluated using the Korean Quality of recovery– 15 (QoR– 15K) score, 0 to 150.

Statistical differences were verified using Wilcoxon rank sum test.

*The crude p-value of multiple comparisons was adjusted using Bonferroni correction.

Abbreviations: IQR, interquartile range.

### Gastric fluid volume and other perioperative outcomes

Surgery-related variables were comparable between the groups (Table 3). Estimated gastric fluid volume, gastric acidity, and degree of oral secretion during anesthesia induction were comparable between the two groups. One patient in the CHD group had a gastric volume greater than a complete empty gastric volume of 1.5 mL/kg (which was 1.69 ml/kg), so the operation was delayed by 1 hour until an empty stomach was confirmed by ultrasound. Postoperative incidence of nausea and vomiting and the need for anti-emetics did not vary significantly between the two groups (Table 3). However, the time to flatus was much shorter in the CHD with gum group compared to the CHD-only group (Table 3).

**Table 3:** Perioperative variables.

Parameter	Control group (N = 52)	Gum group (N = 52)	p-value
Extents of Surgery, n (%)			0.143
Ovarian cystectomy	6 (11.5%)	9 (17.3%)	
Salpingo—oophorectomy	10 (19.2%)	9 (17.3%)	
Myomectomy	11 (21.2%)	5 (9.6%)	
Hysterectomy	22 (42.3%)	29 (55.8%)	
Miscellaneous	3 (5.8%)	0 (0.0%)	
Surgical access, n (%)			0.251
Single port laparoscopic surgery	12 (23.1%)	5 (9.6%)	
Dual port laparoscopic surgery	19 (36.5%)	23 (44.2%)	
Conventional laparoscopic surgery	8 (15.4%)	12 (23.1%)	
Robot-assisted laparoscopic surgery	13 (25.0%)	12 (23.1%)	
Anesthetic time (min), median [IQR]	124.5 [99.0–156.5]	113.5 [94.0–142.0)	0.382
Crystalloid (mL), median [IQR]	645 [450–950]	575 [400–850]	0.347
Estimated blood loss (mL), median [IQR]	80 [50–150]	65 [50–150]	0.904
Urine output (mL), median [IQR]	90 [0–150]	60 [0–150]	0.275
Oral secretion during intubation, n (%)			0.102
None	3 (5.8%)	2 (3.8%)	
Mild	37 (71.2%)	46 (88.5%)	
Moderate	12 (23.1%)	4 (7.7%)	
Severe	0 (0.0%)	0 (0.0%)	
Estimated gastric fluid volume (mL/kg), median [IQR]	0 [0–0.45]	0 [0–0.22]	0.158
Gastric acidity (pH), median [IQR]	1.42 [0.73–4.05]	1.95 [1.08–4.72]	0.183
Total opioid in hospital period	3.67 [3.33–5.83]	3.33 [2.67–4.67]	0.104
PONV, n (%)	31 (59.6%)	23 (44.2%)	0.116
Rescue antiemetics, n (%)	24 (46.2%)	18 (34.6%)	0.318
Time to flatus (min), mean (SD)	1636.3 (539.7)	1237.9 (543.2)	0.001*
Length of stay, median [IQR]	2 [1–2]	2 [1–2]	>0.999

Opioid dose was converted to intravenous morphine equivalent dose.

Abbreviations: IQR, interquartile range; SD, standard deviation.

### Patient-rated postoperative quality of recovery

**Fig 3:**
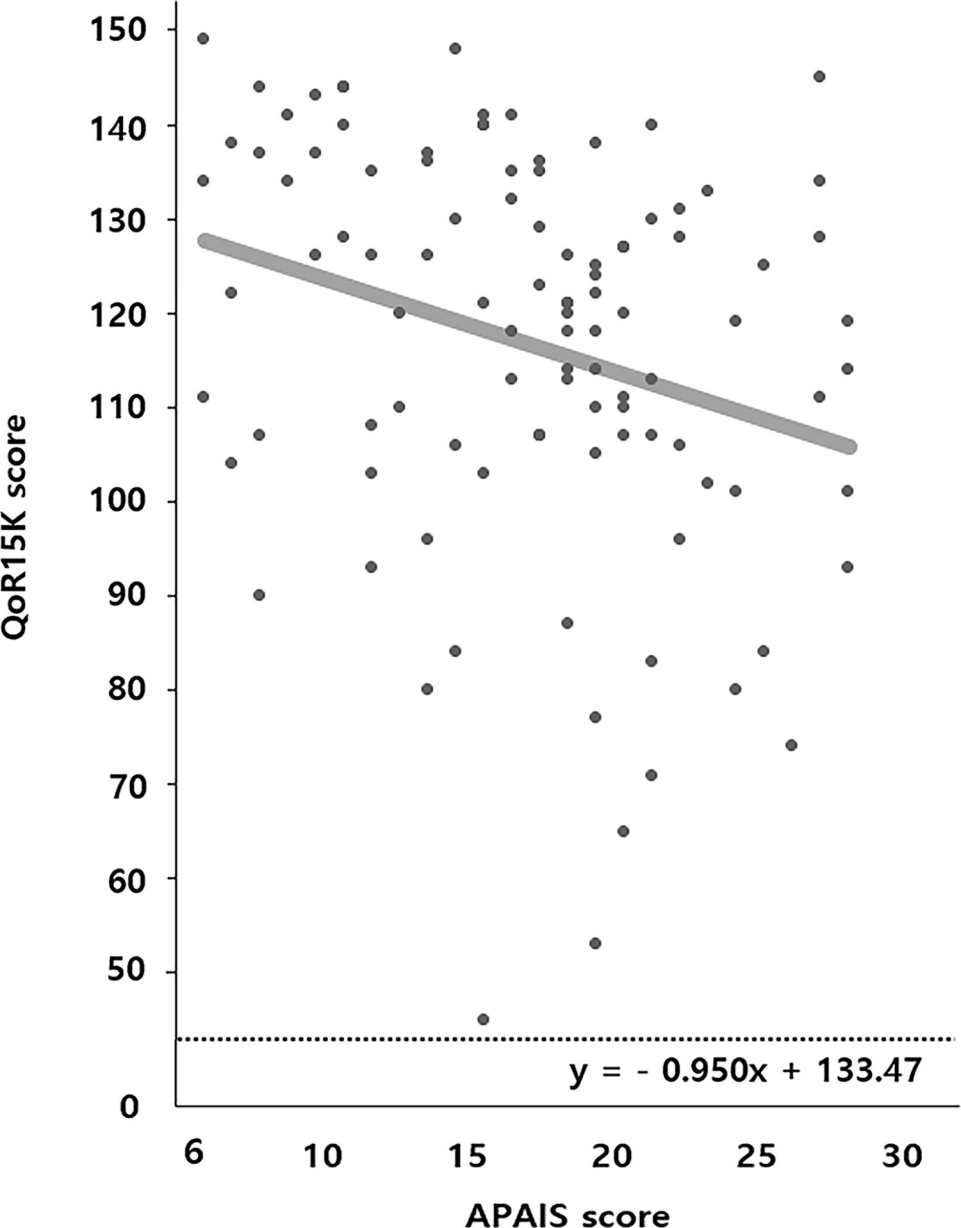
Scatter plot showing the association between QoR-15K and APAIS scores measured in the preoperative holding area.

Postoperative quality of recovery rated by QoR-15K was significantly higher in the CHD with gum group. In linear regression analysis, the postoperative patient-rated QoR-15K score showed a strong negative correlation with the preoperative APAIS score (beta: -0.950, p = 0.001) (Fig 3). However, there was no difference in the length of hospital stay between groups (Table 3).

## Discussion

In this randomized controlled study, we found that preoperative gum-chewing in addition to oral carbohydrate loading with the ERAS fasting protocol resulted in reduced preoperative anxiety without affecting gastric volume or pH values in women patients undergoing gynecologic surgery. In addition, patient-rated postoperative quality of recovery by QoR-15K was also significantly increased in the CHD with gum group.

Chewing gum during preanesthetic NPO is an experimental intervention that conflicts with conventional practice. Although several studies suggest that chewing gum before anesthesia is safe and that chewing gum can be considered an exception to fasting before surgery, there are still vague concerns that chewing gum itself can stimulate oral secretions and gastric juice and affect the amount of gastric contents. The results of this study showed that chewing gum with oral carbohydrate loading was not associated with the volume or acidity of the gastric contents. Regarding oral secretion, there was no significant difference between the groups. Therefore, we confirmed that preanesthetic gum chewing with carbohydrates did not increase the risk of pulmonary aspiration or regurgitation of gastric contents.

APAIS is a validated tool for assessing preoperative anxiety, and a cutoff point of 11 on the anxiety scale was regarded as sufficient for identifying anxious patients [21]. Consistent with previous studies [24,25]. mean preoperative anxiety increased as the surgery schedule approached in the present study; based on a cutoff value of 11 on the anxiety scale, the incidence of anxious participants increased from 47.2% at baseline to 65.7% in the preoperative holding area. APAIS score on the surgery day increased by 17.3% overall compared to baseline, by 24.7% in the CHD group and 10.6% in the CHD with gum group.

Our previous work demonstrated that allowing gum chewing during prolonged preoperative fasting alleviated anxiety [26]. After that study, we launched an institutional ERAS program with the implementation of minimized preoperative fasting and oral carbohydrate loading. Oral carbohydrate loading reduces the postoperative stress response and alleviates patient anxiety, possibly due to an increased plasma serotonin level [27]. We sought to determine whether the addition of gum-chewing to oral carbohydrate loading had still anxiolytic benefits without altering gastric contents. We found that preoperative anxiety level was lower in patients who chewed gum with carbohydrate loading than in the gum chewing plus conventional fasting approach of the previous study, and gum had an anxiolytic effect when added to oral carbohydrate loading. These additional beneficial effects of gum chewing shown in our study can be explained based on the effect of mastication itself. Niwa et al. reported that mastication suppressed stress-induced brain activation [28]. Chewing during stressful conditions lowered the plasma level of stress hormones (catecholamine and adrenocorticotropic hormone) via hypothalamus-pituitary axis suppression [28,29]. Moreover, as a distraction technique for relieving preoperative anxiety, repetitive behaviors may have anxiolytic effects.

In clinical practice, chewing gum is generally recommended only in the postoperative period to facilitate recovery of gastrointestinal motility. The results in the present study showed a shorter time to flatus in the CHD with gum group, and this result may be explained by more vigorous gum-chewing behavior in the postoperative period in the preoperative gum-allowed group. Chewing gum in the postoperative period was recommended in both groups, but patients in the CHD with gum group may have not hesitated to chew gum in the postoperative period. Chewing gum, which simulates sham feeding, was proven to promote gut motility via cephalic-vagal stimulations [30,31]. Furthermore, stereoisomer sugars contained in sugar-free gum are not readily absorbed and act as a laxative [31,32], thus stimulating bowel movement and facilitating flatus.

Although other items of preoperative discomfort were not significantly different between groups, the degree of hunger and unpleasantness in the CHD with gum group was significantly lower than in the CHD group. Furthermore, postoperative quality of recovery rated by QoR-15K was significantly higher in the CHD with gum group. Therefore, chewing gum during pre-anesthetic fasting is a practical and safe strategy that is easy to incorporate into clinical practice and has various benefits such as preoperative anxiety relief, facilitating postoperative bowel recovery, and promoting patient well-being.

This study has certain strengths. First, a wide range of data was collected prospectively by blinded clinicians. Anesthesia, postoperative care, and chewing gum data collection were performed according to preplanned standardized protocols, thus eliminating possible confounders. Second, unlike other studies that dealt with the specific effect limited to bowel recovery, this study explored the possible benefits of chewing gum in a multifaceted and detailed manner. In addition, the effects of gum chewing were evaluated comprehensively to include patient-centered outcomes.

Despite these strengths, the present study also has several limitations. Due to the nature of the study design, this study lacked blinding of the patients. Second, patients may have been anxious since their surgery was confirmed or scheduled, and thus the period of gum chewing might have been short relative to the anxiety period. Finally, it is difficult to generalize the results of our study to patients with increased pulmonary aspiration risk, as these patients were excluded.

In conclusion, the addition of gum chewing to oral carbohydrate loading during preoperative fasting was more effective in relieving preoperative anxiety than oral carbohydrate alone in women patients undergoing elective gynecologic surgery. In addition, this novel approach could be helpful to promote patient satisfaction and improve recovery after laparoscopic gynecologic surgery.

## Supporting information

S1 Checklist CONSORT 2010 checklist of information to include when reporting a randomised trial*. (DOC)

S1 Dataset (XLSX)

S1 File (DOCX)

S2 File (DOCX)

## Abbreviations

APAIS: Amsterdam preoperative anxiety and information scale
BMI: body mass index (BMI)
CHD: carbohydrate drink
CSA: cross-sectional area
ERAS: Enhanced Recovery After Surgery
NRS: numeric rating scale
PCA: patient-controlled analgesia
PONV: postoperative nausea and vomiting
QoR-15: Quality of Recovery-15 questionnaire

## References

[1] Jawaid M, Mushtaq A, Mukhtar S, Khan Z. Preoperative anxiety before elective surgery. Neurosciences (Riyadh). 2007; 12:145–8. . 21857597

[2] Eberhart L, Aust H, Schuster M, Sturm T, Gehling M, Euteneuer F, et al. Preoperative anxiety in adults—a cross-sectional study on specific fears and risk factors. BMC Psychiatry. 2020; 20:140. doi: 10.1186/s12888-020-02552-w . 32228525 PMC7106568

[3] Lemos MF, Lemos-Neto SV, Barrucand L, Vercosa N, Tibirica E. [Preoperative education reduces preoperative anxiety in cancer patients undergoing surgery: Usefulness of the self-reported Beck anxiety inventory]. Braz J Anesthesiol. 2019; 69:1–6. doi: 10.1016/j.bjane.2018.07.004 . 30401475 PMC9391836

[4] Chow CH, Van Lieshout RJ, Schmidt LA, Dobson KG, Buckley N. Systematic Review: Audiovisual Interventions for Reducing Preoperative Anxiety in Children Undergoing Elective Surgery. J Pediatr Psychol. 2016; 41:182–203. doi: 10.1093/jpepsy/jsv094 . 26476281 PMC4884908

[5] Guo P, Li P, Zhang X, Liu N, Wang J, Yang S, et al. The effectiveness of aromatherapy on preoperative anxiety in adults: A systematic review and meta-analysis of randomized controlled trials. Int J Nurs Stud. 2020; 111:103747. doi: 10.1016/j.ijnurstu.2020.103747 . 32861206

[6] Nelson G, Altman AD, Nick A, Meyer LA, Ramirez PT, Achtari C, et al. Guidelines for pre- and intra-operative care in gynecologic/oncology surgery: Enhanced Recovery After Surgery (ERAS(R)) Society recommendations—Part I. Gynecol Oncol. 2016; 140:313–22. doi: 10.1016/j.ygyno.2015.11.015 . 26603969

[7] Sketchley-Kaye K, Jenks R, Miles C, Johnson AJ. Chewing gum modifies state anxiety and alertness under conditions of social stress. Nutr Neurosci. 2011; 14:237–42. doi: 10.1179/1476830511Y.0000000017 . 22053754

[8] Yaman-Sozbir S, Ayaz-Alkaya S, Bayrak-Kahraman B. Effect of chewing gum on stress, anxiety, depression, self-focused attention, and academic success: A randomized controlled study. Stress Health. 2019; 35:441–6. doi: 10.1002/smi.2872 . 31125164

[9] Yildizeli Topcu S, Akgun Kostak M, Semerci R, Guray O. Effect of Gum Chewing on Pain and Anxiety in Turkish Children During Intravenous Cannulation: A Randomized Controlled Study. J Pediatr Nurs. 2020; 52:e26–e32. doi: 10.1016/j.pedn.2019.12.007 . 31889572

[10] Bouvet L, Loubradou E, Desgranges FP, Chassard D. Effect of gum chewing on gastric volume and emptying: a prospective randomized crossover study. Br J Anaesth. 2017; 119:928–33. doi: 10.1093/bja/aex270 . 29077816

[11] Poulton TJ. Gum chewing during pre-anesthetic fasting. Paediatr Anaesth. 2012; 22:288–96. doi: 10.1111/j.1460-9592.2011.03751.x . 22171675

[12] Schoenfelder RC, Ponnamma CM, Freyle D, Wang SM, Kain ZN. Residual gastric fluid volume and chewing gum before surgery. Anesth Analg. 2006; 102:415–7. doi: 10.1213/01.ane.0000189218.07293.6e . 16428535

[13] Smith I, Kranke P, Murat I, Smith A, O’Sullivan G, Soreide E, et al. Perioperative fasting in adults and children: guidelines from the European Society of Anaesthesiology. Eur J Anaesthesiol. 2011; 28:556–69. doi: 10.1097/EJA.0b013e3283495ba1 . 21712716

[14] Garcia AKA, Furuya RK, Conchon MF, Rossetto EG, Dantas RAS, Fonseca LF. Menthol chewing gum on preoperative thirst management: randomized clinical trial. Rev Lat Am Enfermagem. 2019; 27:e3180. doi: 10.1590/1518-8345.3070.3180 . 31596415 PMC6781380

[15] Desgranges FP, Chassard D, Bouvet L. Pre-operative gum chewing: forbidden, allowed or recommended. Anaesthesia. 2019; 74:539. doi: 10.1111/anae.14616 . 30847912

[16] Nelson G, Bakkum-Gamez J, Kalogera E, Glaser G, Altman A, Meyer LA, et al. Guidelines for perioperative care in gynecologic/oncology: Enhanced Recovery After Surgery (ERAS) Society recommendations-2019 update. Int J Gynecol Cancer. 2019; 29:651–68. doi: 10.1136/ijgc-2019-000356 . 30877144

[17] Birenbaum A, Hajage D, Roche S, Ntouba A, Eurin M, Cuvillon P, et al. Effect of Cricoid Pressure Compared With a Sham Procedure in the Rapid Sequence Induction of Anesthesia: The IRIS Randomized Clinical Trial. JAMA Surg. 2019; 154:9–17. doi: 10.1001/jamasurg.2018.3577 . 30347104 PMC6439856

[18] Perlas A, Mitsakakis N, Liu L, Cino M, Haldipur N, Davis L, et al. Validation of a mathematical model for ultrasound assessment of gastric volume by gastroscopic examination. Anesth Analg. 2013; 116:357–63. doi: 10.1213/ANE.0b013e318274fc19 . 23302981

[19] Song IK, Kim HJ, Lee JH, Kim EH, Kim JT, Kim HS. Ultrasound assessment of gastric volume in children after drinking carbohydrate-containing fluids. Br J Anaesth. 2016; 116:513–7. doi: 10.1093/bja/aew031 . 26994229

[20] Van de Putte P, Perlas A. Ultrasound assessment of gastric content and volume. Br J Anaesth. 2014; 113:12–22. doi: 10.1093/bja/aeu151 . 24893784

[21] Moerman N, van Dam FS, Muller MJ, Oosting H. The Amsterdam Preoperative Anxiety and Information Scale (APAIS). Anesth Analg. 1996; 82:445–51. doi: 10.1097/00000539-199603000-00002 . 8623940

[22] Shin WJ, Kim YC, Yeom JH, Cho SY, Lee DH, Kim DW. The Validity of Amsterdam Preoperative Anxiety Information Scale in the Assessment of the Preoperative Anxiety—Compared with hospital anxiety depression scale and visual analogue scale. Korean J Anesthesiol. 1999; 37:179–87. 10.4097/kjae.1999.37.2.179.

[23] Yoon S, Joo H, Oh YM, Lee J, Bahk JH, Lee HJ. Validation and clinical utility of the Korean version of the Quality of Recovery-15 with enhanced recovery after surgery: a prospective observational cohort study. Br J Anaesth. 2020; 125:614–21. doi: 10.1016/j.bja.2020.06.040 . 32703550

[24] Jiwanmall M, Jiwanmall SA, Williams A, Kamakshi S, Sugirtharaj L, Poornima K, et al. Preoperative Anxiety in Adult Patients Undergoing Day Care Surgery: Prevalence and Associated Factors. Indian J Psychol Med. 2020; 42:87–92. doi: 10.4103/IJPSYM.IJPSYM_180_19 . 31997870 PMC6970311

[25] Kumar A, Dubey PK, Ranjan A. Assessment of Anxiety in Surgical Patients: An Observational Study. Anesth Essays Res. 2019; 13:503–8. doi: 10.4103/aer.AER_59_19 . 31602069 PMC6775825

[26] Bang YJ, Lee JH, Kim CS, Lee YY, Min JJ. Anxiolytic effects of chewing gum during preoperative fasting and patient-centered outcome in female patients undergoing elective gynecologic surgery: randomized controlled study. Sci Rep. 2022; 12:4165. doi: 10.1038/s41598-022-07942-6 . 35264684 PMC8907183

[27] Wurtman RJ, Wurtman JJ. Do carbohydrates affect food intake via neurotransmitter activity? Appetite. 1988; 11 Suppl 1:42–7. . 2903717

[28] Niwa M, Hiramatsu I, Nakata F, Hamaya C, Onogi N, Saito K. Functional Significance of Stress-relieving Act of Chewing and it Effect on Brain Activation by Strees. J Jpn Assoc Rural Med. 2005; 54:661–6. 10.2185/jjrm.54.661.

[29] Azuma K, Zhou Q, Niwa M, Kubo KY. Association between Mastication, the Hippocampus, and the HPA Axis: A Comprehensive Review. Int J Mol Sci. 2017; 18:1687. doi: 10.3390/ijms18081687 . 28771175 PMC5578077

[30] Pera P, Bucca C, Borro P, Bernocco C, De LA, Carossa S. Influence of mastication on gastric emptying. J Dent Res. 2002; 81:179–81. 10.1177/0810179 . 11876271

[31] Jernigan AM, Chen CC, Sewell C. A randomized trial of chewing gum to prevent postoperative ileus after laparotomy for benign gynecologic surgery. Int J Gynaecol Obstet. 2014; 127:279–82. doi: 10.1016/j.ijgo.2014.06.008 . 25147092

[32] Tandeter H. Hypothesis: hexitols in chewing gum may play a role in reducing postoperative ileus. Med Hypotheses. 2009; 72:39–40. doi: 10.1016/j.mehy.2008.06.044 . 18783895

