# Supplementary material for: The effect of adding chewing gum to oral carbohydrates on preoperative anxiety scores in women undergoing gynecological surgery: A randomized controlled study
Source: PLoS One. 2023 Apr 25;18(4):e0283780. doi: 10.1371/journal.pone.0283780 (PMC10129008; doi:10.1371/journal.pone.0283780)
Supplement: S1 File — (DOCX) [file pone.0283780.s003.docx]

**【임상시험 계획서】**

**1. 임상시험의 명칭 및 단계**

ERAS protocol으로 부인과 복강경 수술을 받는 환자에서, 마취 전 금식 기간 내 껌 씹기가 환자 불안도 및 수술 후 회복에 미치는 영향에 대한 무작위 비교 연구

(Effects of chewing gum during pre-anesthetic fasting on preoperative anxiety and postoperative recovery in patients undergoing laparoscopic gynecological surgery with ERAS protocol : randomized controlled study)

**2. 임상시험 실시 기관명 및 주소**

삼성서울병원 마취통증의학과

서울특별시 강남구 일원로 81, 삼성서울병원 마취통증의학과

**3. 임상시험책임자, 담당자 및 공동연구자의 성명 및 직명**

1) 임상시험책임자: 마취통증의학과 조교수 민정진

2) 연구담당자: 마취통증의학과 임상강사 방유정

**4. 임상시험의 목적 및 배경**

1) 연구목적:

(1) 1차 목적: ERAS protocol에 따라 복강경으로 부인과 수술을 받는 환자에서, 마취 전 금식 기간 내 껌을 사용한 거짓 식이가 수술 전 환자의 불안도와 금식과 관련한 불편한 증상에 미치는 영향을 알아본다..

(2) 2차 목적: 복강경으로 부인과 수술을 받는 환자에서, 두 군간에 위장관 기능 회복, 위장관합병증 발생, 수술 후 회복을 비교하고자 한다.

2) 연구배경

전신 마취하 수술을 받는 환자들의 경우 gastric fluid regurgitation, pulmonary aspiration등의 complication을 예방하기 위한 목적으로 ASA guideline에 따라 light meal 6시간 clear liquid 2시간의 금식을 엄수하도록 되어있다. 현재 여러 방면의 수술 환자군에서 수술 후 조기회복을 위하여 ERAS setting이 도입되었고, 이 가이드라인에 따라 맑은 액체류를 마취 2시간 전까지 충분히 섭취하도록 하고 탄수화물 음료를 금식기간동안 투여하도록 권고된다. 수술 전 carbohydrate의 투여로 금식으로 인한 catabolic state, 회복의 지연 등의 부작용을 막을 수 있으며, 이를 경구로 투여함으로써 환자의 만족도 및 well - being sense를 향상시킬 수 있기때문이다. 경구로 Carbohydrate를 투여하는 방법으로 탄수화물 음료를 수술 전 2시간까지 800ml에서 400ml정도 투여하는 것이 표준 요법으로 권장되고 있다. ERAS society에서는 수술 전 금식 기간의 껌 씹기 역시 긍정적인 효과가 있어서 추천할 수 있다는 의견을 제시하고 있다. 수술 전 금식 기간의 껌 씹기는 수술을 앞둔 환자에게 위 내 용적 및 산도에 영향을 주지 않고 환자의 불안감을 낮추며 금식으로 인한 부작용을 감소시킨다는 연구 결과들이 최근 발표되었다. 특히 본원 산부인과 환자를 대상으로 하여 수술 전 껌 씹기는 수술 전 불안감을 낮추고 수술 후 회복을 향상시키는데 유의하였다는 연구가 최근 수행되었다(SMC 2019-05-168-001). 이 연구 결과 역시 기존 연구 결과와 유사하게 어떠한 부작용 사례도 보고되지 않았다.

현재 본원 산부인과에서는 부인암센터의 elective surgery를 받기로 예정된 환자에게 ERAS protocol을 시범적으로 적용하기로 결정하였고 이에 따라 수술 전 금식시간에 탄수화물 음료를 복용하게 된다. 본 연구는 수술전 금식시간내에 껌씹기의 불안감 경감효과에 대한 연구의 (SMC 2019-05-168-001) 후속 연구로서, 산부인과의 변경된 진료 프로토콜에 참가하는 환자를 대조군으로 설정하고 수술 전 금식 기간에 껌을 사용한 sham feeding을 추가적으로 시행함으로써 수술 전 불안감과 불편감을 경감시켜주는 효과가 있는지 알아보고자 한다. 이외에 각 그룹간에서 수술 후 bowel function의 recovery및 식이 재개, 수술후 회복 및 well - being sense에 차이가 있는지 비교하려 한다.

3) 연구설계; 전향적, 무작위 배정

**5. 임상시험용 식품**

1) 식용 껌

(1) 식품명: 자일리톨 껌

(2) 성분명: D-말티톨, 자일리톨, 껌 기초제,

이소말트, 말티톨 시럽, 아라비아검, 합성향료, 감미료

2) 식용 탄수화물 음료

(1) 식품명: 뉴케어 노엔피오

(2) 성분명: 정제수, 덱스트린, 결정과당, 산도조절제,

합성 착향료, 수크랄로스, 말토덱스트린, 토마틴

**6. 대상 질환 및 피험자의 선정기준, 제외 기준**

1) 선정기준

(1) ASA class I-Ⅲ 성인 환자 (만19세 이상 70세 미만)

(2) Laparoscopic gynecologic surgery를 받는 환자

2) 제외 기준

(1) 소아 (19세 미만)

(2) 고령 (70세 이상)

(3) 임산부, 수유부

(4) 생체 징후가 불안정한 환자

(5) 폐 흡인 가능성이 높은 환자 [3]

BMI 30이상, gastrooesophageal reflux, renal failure, oesophageal strictures, achalasia, enteropathies, diabetes mellitus, 위장관 종양의 병력이 있는 자,

(6) Any medication that could affect GI motility

(7) 간, 신장 질환자

(8) 응급 수술.

(9) 연구 참여에 동의하지 않는 환자

3) 취약한 대상자 모집 시, 보호 방안

모든 연구 대상자는 1일전에 동의를 득하고, 설명에 30분 이상 충분한 시간을 할애할 것이다. 이해력과 판단력이 저하된 대상자의 경우 보호자(또는 대리인)의 추가적인 동의를 받는 등의 추가적인 보호조치를 할 것이다.

**7. 목표한 피험자 수 및 근거:**

1) 일차 가설: 수술전 금식기간에 경구용 탄수화물 식품을 복용하는 군의 불안도보다 수술전 금식 기간에 경구용탄수화물 식품을 복용하면서 추가로 껌을 씹는 군의 불안도가 낮다.

2) 기존 연구 결과[4]로부터 탄수화물 음료 복용시 수술직전에 VAS로 측정한 불안도의 평균값이 34.83 이고 표준편차가 14.8이다. 또 여성의 경우 수술전 불안도의 평균값이 15%정도 더 높은것으로 보고되었다[5]. 이 연구 결과를 바탕으로 수술직전 여성 환자군의 수술전 불안도의 VAS가 39.31 ± 17.04로 보정하였고 두군간 VAS- anxiety가 25%감소 하면 껌을 씹는 것이 불안도를 경감시킨다고 할 때, 유의수준 5%, 검정력 80%하 에서 각 군당 47명이 필요하다. 탈락률을 10%로 예상하여 각군당 52명 도합 104명의 환자가 필요하다.

**8. 임상시험기간**

임상시험 승인일부터 1년

**9. 임상시험방법**

1) 시험방법 전체 flow

1. 환자는 수술 전날 무작위방법으로 시험군과 대조군에 배정되며, 시험에 참여하지 않는 산부인과 주치의에게 주의사항을 듣고 노엔피오를 배부 받게 된다. 양군 모두 수술 전일 오후 3시부터 고형식을 금하고 clear liquid만 허용하며 수술 전3시간부터 물도 금한다.
2. 대조군 (group C)의 경우 본원 산부인과 프로토콜 대로 수술 전 금식을 엄수하면서 석식 대신 노엔피오 두캔을 복용, 첫 수술 환자 오전 5시 노엔피오 1캔, 첫 수술을 제외한 오전 수술 환자는 수술 3시간전 노엔피오 1캔, 오후 수술 환자는 오전 7시, 수술 3시간 전 각각 노엔피오 1캔을 복용하게 된다.
3. 시험군 (Group G)의 경우 본원 산부인과 ERAS 프로토콜 대로 대조군과 같은 방식으로 수술 전 금식을 엄수하면서 탄수화물 음료를 복용하는 한편 금식시간내에 제공된 껌을 자유롭게 씹을 수 있다.
4. 두 환자군 사이에 금식 시간 중 제공되는 껌의 유무 이외에 다른 중재는 없으며 이후 마취 및 마취 전 검사 과정은 동일하게 진행된다.
5. 껌은 수술실로 이송 직전 제거하고 수술실로 이동하게 되며, 수술실 담당 간호사가 수술실 대기실에서 환자 확인 과정 중 제거 유무를 2차로 확인한다.
6. 수술실 대기실에서 실제 마취를 담당하게 될 마취과 의사 또는 마취과 간호사가 환자의 불안도대하여 설문조사를 시행한다.
7. 모든 환자는 입실 후 통상적인 마취 절차에 따라 full monitoring(EKG, NIBP, SpO2) 후에 O2로 3분간 pre-oxygenation을 한다. Pre oxygenation기간 중 연구 담당자인 방유정이 복부 초음파를 시행하여 위 전정부(antrum)의 용적을 측정한다.
8. 그후 담당 마취의가 3차로 구강내 껌 유무를 확인한 뒤 Propofol과 remifentanil, rocuronium의 TIVA 약제를 사용하여 induction을 시행한다. 마취의가 intubation을 시행하면서 oral secretion의 정도를 평가한다.
9. 마취 유도 종료 직후 모든 환자에게 통상적인 마취 절차에 따라 esohphageal temperature probe (상품명: ST probe)를 삽입한다. ST tube 의 gastric port를 통하여 gastric fluid를 natural drain시켜 얻은 후 pH meter로 acidity 를 평가한다. pH만 기록한 뒤 위 내용물은 바로 수술실 내에서 폐기한다.
10. 수술 진행과 마취 유지에 있어서 이후 과정은 특별한 중재없이 진행한다. 총 마취 시간, 수술 시간, 마취 중 사용된 약제 및 vital sign등은 의무기록을 통하여 수집한다. (증례보고지별첨)
11. 퇴원일까지 회복실 및 병동에서 환자의 수술 후 회복과 위장관 합병증에 대한 증상을 의무 기록과 환자 설문지를 통하여 수집한다. (설문지 별첨1)

2) 무작위 및 눈가림 방법

산부인과 ERAS 센터에서 폐흡인의 저위험군에 한하여 ERAS protocol을 적용할 환자를 선정하게 된다. ERAS protocol 을 적용받는 환자들 중에서 block size 2로 하는 random permuted block design에 의해 무작위 배정 리스트를 작성하여 1:1의 비율로 각 군에 배정된다. 무작위 배정 정보는 평가자에게는 해당 리스트가 공개되지 않고 그 밖의 연구자만 알 수 있도록 관리된다. 수술실에 입실한 후 대조군과 시험군 모두 동일한 스터디 프로토콜에 따라 진행하게 되며 위 내 용적 측정 및 산도의 기록은 모두 연구 책임자에 의해 기록한다. 수술 직전 불안도 및 금식과 관련한 불편한 증상은 환자의 자가 보고를 바탕으로 연구에 참여하지 않는 담당 마취의가 기록한다. 수술 후 위장관 운동 회복 및 수술 후 회복에 대한 설문지는 독립적인 산부인과 병동 간호사와 주치의의 안내를 받아 환자가 직접 작성한다.

**10. 관찰 항목 및 방법**

1) Patient characteristics: Age, sex, height, weight, BMI, ASA class and other comorbidities,

smoking, alcohol

2) Gastric fluid volume & pH

환자에게 Rt. Lateral dequbitus position을 취하게 한 후 Abdominal ultrasonography로 antrum의 직경을 확인하고 다음과 같은 공식을 사용하여 gastric fluid level을 확인한다.

* volume (ml) = 27 + 14.6 9 * antral area (cm²) – 1.289 * age (year)

Induction이 끝나고 나서 환자에게 거치된 esophageal probe를 통해 gastric fluid를 채취한 후

pH meter를 사용하여 pH를 측정한다.

3) Pre-operative anxiety

(1) Anxiety with VAS

(2) Anxiety with APAIS score


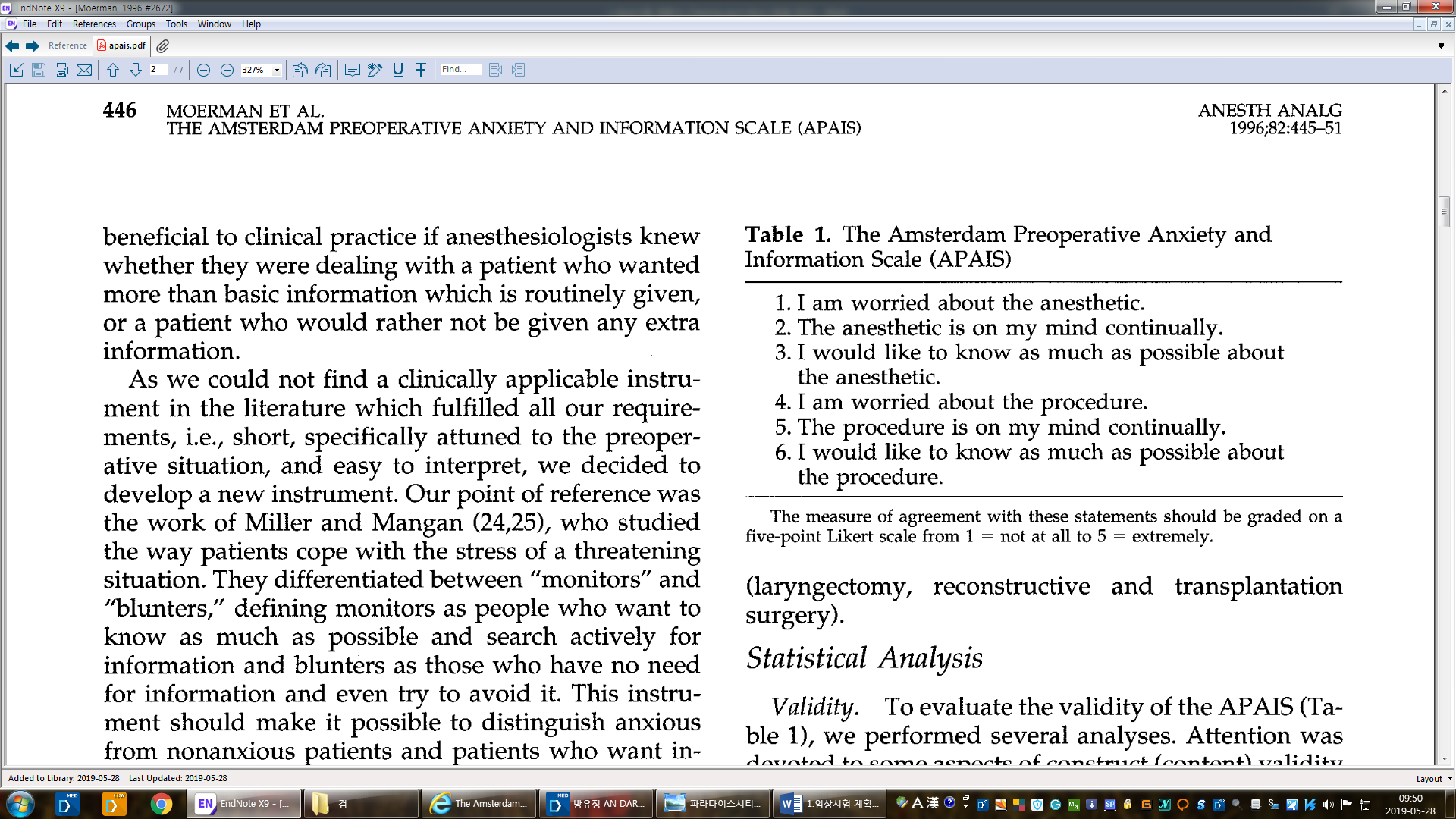


APAIS score는 수술 전 환자의 불안도를 측정하기 위해 개발된 문항으로서 여러 연구에서 그 유효성이 검증되었다. APAIS score 는 6가지 문항에 1점부터 5점까지의 점수를 총합하여 계산한 것으로 점수가 클수록 불안도가 높음을 시사하고, 총점 13점이상이면 불안 수가 높은 것으로 간주한다.

(3) Symptom related to anxiety ^:^

, 금식 시간에 껌을 씹는 것이 금식 시간을 견디는데 도움이 되었는지, 집중하기 어려움Hunger, thirst, dry mouth, fatigue, headache, nausea 등 불안감과 관련된 증상을 각 항목별로 0에서 10점 사이의 점수로 설문한다. (설문지 별첨1)

4) Recovery or bowel function

1. Post- operative nausea & vomiting
2. Flatus expulsion, defecation time in postoperative time, time of resume feeding
3. Intra hospital bowel complication (ileus, dyspepsia, abdominal pain)

5) QoR15

QoR 15는 수술후 환자의 회복정도를 각 항목에 0점에서 10점까지 점수로 설문하여 총합으로 계산한 것으로 점수가 클수록 양질의 회복을 시사한다.

6) 그 외 관찰 항목

(1) intubation시점에서 oral secretion의 양정도 (none/ mild/ moderate/severe)

(2) 마취 중 사용된 anesthetic drug의 total dose, 수액의 양

(3) 총 마취 시간 및 수술시간

(4) Intra hospital period에서 사용된 opioid의 총량

7) 일정표

**11. 예측 부작용 및 사용상의 주의사항**

알려진 바에 따르면 전신마취에서 pulmonary aspiration의 incidence는 대략 1:7000로 매우 희소하고, aspiration으로 인한 및 death는 1:100000로 매우 희소하다[6]. 껌을 씹는 중재적 행위에서 가장 염려되는 것은 위 내 용적의 증가 및 그로 인해 흡인의 위험성이 증가하지 않는지 하는 부분이다. 그러나 본 계획서 4. (2)에서 상술한 바와 같이 이전연구에서 성인과 소아에서 수술 전 껌을 씹었을 때 위산 분비 및 위 내 용적이 크게 증가하지 않는다는 결과가 있다[7, 8]^.^ 건강한 자원자를 대상으로 조사직전까지 껌을 씹은 군과, 2시간전 물 250ml를 마신 군에서 위내 용적 및 산도를 비교한 결과 두 군간에 전혀 차이가 없다는 연구 결과가 있다. 유럽 마취과 학회(ESA)에서 발표된 가이드라인에서도 수술 직전 껌을 씹었다는 이유로 수술과 마취를 지연시키지 않으며, 껌 씹기가 환자의 aspiration risk를 높이지 않는다고 확정 지었다^.^ 학계 에서는 2019년 Anesthesia 지에 실린 전문가의 review처럼 preoperative fasting period에 껌 씹기를 권장해야 할 것이라는 의견도 제시되고 있다. 특히 본원 산부인과 환자를 대상으로 금식 기간에 껌 씹기를 하였을 때 고전적인 금식을 환자와 비교하여 위내 용적, 위산도, 침 등의 분비물에 있어서 차이가 없었다.

본 연구에서는 마취 전날 환자 정보를 조사하고, 폐 흡인의 risk가 높은 사람 (비만, 위장관 질환, DM, 간신장질환자)은 본 연구에서 미리 exclusion할 것이므로 pulmonary aspiration의 risk는 증가 하지 않는다. 그러나 혹시 모를 흡인 가능성에 대하여 대처하기 위한 방법으로 본 연구에서는 추가로 1명의 마취과전문의가 Sellick maneuver를 사용하여 보조하고, 만일의 event에 대처할 수 있도록 한다.

수술실 내에서 시행되는 복부 초음파 검사는 연구를 위해 추가되는 검사로서, 비침습적인 방법으로 복부 장기를 관찰할 수 있는 방법으로 특별한 부작용은 없으나 환자의 명치 부위 피부에 초음파용 전도 겔을 바르고, 1~2분정도 누워있어야 하는 불편함이 있다. 그러나 기존의 수술에서 위 초음파 없이 intubation을 하는 것보다 위내 고형물의 유무, 위 용적의 정보를 미리 담당 마취과 의사에게 제공할 수 있다는 장점이 있다.

그 이외 Esophageal probe 를 통해 gastric fluid 채취로 인한 추가 위험의 상승은 없다. 전신 마취를 하는 동안 Esophageal probe를 삽입하는 것은 표준 진료절차이다. Esophageal probe에는 stethoscope및 temperature sensor 등의 기능이 있어 수술 중 환자의 중심 체온을 monitor하게 된다. 본원에서 사용하는 Esophageal probe (상품명 ST tube)는 Gastric suction port가 있어서 수술 중 진료과의 요청 또는 마취과의 필요에 따라 위장내 감압, 위액의 drainage 등이 가능하다. Acidity의 분석을 위해서는 기존 표준 마취 절차로서 거치된 Esophageal probe의 gastric suction port에서 소량 (1~2방울)만 채취하면 충분하다. 위액의 채취로 인해 추가적인 위험도의 상승은 예상되지 않으며, 채취된 위액은 수술실 내에서 분석 후 바로 폐기한다.

**12. 중지/탈락 기준**

1) 마취 전후 hemodynamic unstable state인 경우

2) 이전에 진단되지는 않은 환자가 마취 약제에 대한 알레르기 반응을 보이는 경우

3) 대상자의 자발적인 동의 철회

4) 연구 담당의사의 지시를 따르지 않음

5) 연구 참여와 관계 없는 중대한 질환이 발생함

6) 담당의사가 귀하에게 있어 연구가 최선의 방법이 아니라고 결정함

**13. 효과 평가기준, 평가방법 및 해석 방법 (통계분석방법)**

1) Primary outcome: Anxiety with VAS APAIS score, Symptom related to anxiety

2) Secondary outcome: Flatus expulsion, defecation time in postoperative time, time of resume feeding, Intra hospital bowel complication, gastric fluid volume & pH, oral secretion at intubation time

3) Analysis

(1) Demographic factor 및 clinical factor

두 군간 비교 검정은 연속형변수의 경우 two-sample t-test 또는 Wilcoxon’s rank sum test로, 범주형변수는 Chi-square test, Fisher’s exact test로 수행한다.

(2) Primary endpoint

기술 통계는 평균, 중위수, 표준편차, 사분위 범위 등으로 제시하며, 시험군과 대조군간 VAS와 APAIS 점수를 two-sample t-test로 비교 검정한다.

(3) Secondary endpoint

연속형변수는 평균, 중위수, 표준편차, 사분위 범위 등으로 기술 통계를 제시하며 two

sample t-test 또는 Wilcoxon’s rank sum test로 두 군간 차이를 검정한다. 범주형변수는 빈도, 비율로 기술 통계를 제시하며 Chi-square test 또는 Fisher’s exact test로 두 군간 비교 검정한다. 두 군간 차이가 있는 인구학적, 임상적 변수가 있다면, multiple linear regression, multiple logistic regression으로 이 변수들을 보정하여 두 군간 일차 변수 및 이차 변수가 차이가 있는지를 검정한다.

**14. 부작용을 포함한 안전성의 평가기준, 평가방법 및 보고 방법**

본 연구는 금식 기간 껌씹기라는 중재가 있는 전향적 연구이다. 예측되는 부작용은 이전 연구결과에 따라 고전적 금식 후 마취 방법과 비교하여 부작용은 차이가 없으므로 위 내용물의 폐 흡인은 예측되지 않는다. 그러나 마취 유도시 구강내 껌으로 인한 기도 폐색의 우려가 있기 때문에 이에 대하여서는 마취 전 단계에서 3번에 걸쳐 껌을 제거했는지 확인 평가 할 것이다. 이후 마취 과정에서도 video assist device로 환자의 기도를 관찰하고 껌의 기도 폐색을 평가 후 기관삽관을 할 것이다. 본 임상시험에 참여하는 모든 환자군에서 수술실 입실 직후, 수술 중, 회복실 퇴실 시점까지 지속적으로 환자의 혈압, 맥박, 산소 포화도, 호흡수 등의 활력 징후들을 철저히 감시, 평가할 것이다. 이 후 수술 후 72시간까지 의료진이 방문하여 부작용 여부를 조사할 것이다. 본 시험 기간 중 임상시험책임자 및 담당자는 대상자의 안전에 만전을 기할 것이며 중대한 이상반응 발생시에는 시험을 중지하고 신속하고 적절한 조치를 취하여 이상반응을 최소화할 것이다. 그리고 중대한 이상반응 본 기관 지침에 따라 IRB에 보고할 예정이다.

**15. 피험자동의서양식**

별도 양식 첨부

**16. 피해자보상에 대한 규약**

1) 보상기준:

임상연구에 따른 중재(개입)의 이상반응에 대해여 당사자들간에 미리 합의한 보상액 또는 조치가 있는 경우, 당해 기준에 따라 이를 보상한다. 그 외의 경우에는 신체적인 손상의 정도, 성격, 지속기간, 유사사례 등을 종합적으로 고려하여 당사자들간의 합의한 보상 방법에 따라 이를 보상한다. 당사자들간에 전항의 합의가 이루어지지 아니한 경우에는 법원의 판결 및 이에 준하는 결정의 확정 내용에 따라 보상한다.

1. 보상재원:

원내보상기준을 이용한다. (본 임상시험과 관련된 손상이 발생하여 응급조치가 필요할 경우, 삼성서울병원에서 행해지는 처음 24시간 동안의 응급조치에 필요한 치료비에 대해서 삼성서울병원에서 부담한다. 부작용 발생의 경우는 이미 알려진 치료방법으로 치료 받게 된다.)

**17. 증례기록서양식**

별도 양식 첨부

**18. 임상시험 후 피험자의 진료 및 치료 기준**

특별한 진료 및 치료가 필요하지 않을 것으로 기대한다. .

**19. 기타 임상시험을 안전하고 과학적으로 실시하기 위하여 필요한 사항**

이전 연구에서 마취된 상태로 껌이 발견된 사례가 1case 있어 껌의 흡인을 유의해야한다. 껌은 수술실로 이송 직전 제거하는데 1차 확인은 병동 간호사가 하게 되고 수술실 대기실에서 수술실 간호사가 제거 유무를 2차로 확인한다. 마취 약제를 투여하기 전 담당 마취의가 3차로 껌의 제거 유무를 확인하여, 껌 흡인 사고를 방지하도록 한다. 본 임상시험에 참여하는 환자는 마취 유도 전부터 회복실 퇴실 시점까지 지속적으로 환자의 혈압, 맥박, 산소 포화도, 호흡수 등의 활력 징후들을 감시, 평가할 것이다. 이외 본 연구에 있어서 예상치 못한 안정성 문제가 발견될 시에는 임상시험을 즉시 중지하고 중대한 이상 반응 보고서 양식에 따라 보고할 예정이다.

**20. 피험자의 안전 보호에 관한 대책**

본 연구에서 획득된 자료는 연구 외의 다른 목적으로 사용되지 않을 것이며 증례 기록지에 환자의 개인정보 노출을 방지하기 위해 숫자로 코드화하여 기록하며 피험자의 신원을 파악할 수 있는 기록은 비밀로 보장될 것이다.
