# Supplementary material for: The effect of adding chewing gum to oral carbohydrates on preoperative anxiety scores in women undergoing gynecological surgery: A randomized controlled study
Source: PLoS One. 2023 Apr 25;18(4):e0283780. doi: 10.1371/journal.pone.0283780 (PMC10129008; doi:10.1371/journal.pone.0283780)
Supplement: S2 File — (DOCX) [file pone.0283780.s004.docx]

【Clinical study protocols】

**1. The name of clinical study and design.**

Effects of chewing gum during pre-anesthetic fasting on preoperative anxiety and postoperative recovery in patients undergoing laparoscopic gynecological surgery with ERAS protocol: A randomized controlled study

**2. Institution**

Samsung medical center, Department of anesthesiology and pain medicine

Irwon-ro 81, Gangnam-gu, Seoul, Republic of Korea

**3. Investigators**

1) Primary investigator: Assistant professor Jeong-Jin, Min

2) Sub investigator: Clinical fellow Yu Jeong, Bang

**4. Study goals and background information**

1) Study goals

(1) Primary objective: This study aims to investigate whether sham feeding with sugar-free gum added to oral carbohydrates during pre-anesthetic fasting would relieve preoperative discomfort and anxiety in a patient undergoing laparoscopic gynecologic surgery.

(2) Secondary objective: Secondary objective is to investigate whether sham feeding with sugar-free gum added to oral carbohydrates during pre-anesthetic fasting would not only relieve preoperative discomfort and anxiety but also promote postoperative bowel function.

2) Rationale & background information

For patients undergoing surgery under general anesthesia, fasting for 6 hours of light meal and 2 hours of clear liquid is required in accordance with the ASA guideline to prevent complications such as gastric fluid regurgitation and pulmonary aspiration. Currently, the ERAS protocol has been introduced for enhanced recovery after surgery in patients undergoing various types of surgery, and it is recommended to allow clear liquids 2 hours before anesthesia and administer carbohydrate drinks during the fasting period. The administration of carbohydrates before surgery can prevent negative effects such as catabolic state and delayed recovery caused by fasting and improve patient satisfaction and well-being sense. The oral carbohydrate recommended to drink 400ml to 800ml up to 2 hours before surgery. The ERAS society suggests that chewing gum during the fasting period before surgery also has a positive effect and can be recommended. We previously studied the anxiolytic effect of chewing gum undergoing gynecologic surgery. (SMC 2019-05-168-001) Chewing gum during preoperative fasting is also thought to facilitate gastrointestinal movements, which will help restore gastrointestinal function. Previous studies have shown that sugar-free gum is not likely to affect the volume and acidity of the stomach immediately after anesthesia compared to patients who did not chew gum, and therefore the risk of regurgitation and pulmonary aspiration is not likely to make a big difference. However, the effect and safety profile of adding chewing gum to oral carbohydrates was not investigated.

We aim to investigate whether sham feeding with sugar-free gum added to oral carbohydrates during pre-anesthetic fasting would not only relieve preoperative discomfort and anxiety but also promote postoperative bowel function.

Currently, the Department of Obstetrics and Gynecology at our institution has decided to launch the ERAS protocol for patients scheduled to undergo elective laparoscopic gynecologic surgery. This study is a follow-up study on the anxiety-reducing effect of chewing gum during the fasting period before surgery. Patients participating in the ERAS protocol were set as a control group and sham feeding using gum was performed during the fasting period in the experimental group. The purpose of this study is to find out if chewing gum plus carbohydrate is effective in reducing anxiety and discomfort before surgery.

3) design: Single-center, randomized controlled study, investigator blinded.

**5. Study food**

1) Gum

(1) Name: Xylichew, Black Licorice (calories 5cal)

(2) Ingredient: Xylitol, gum base, vegetable glycerin, natural anise & licorice flavors, gum arabic, sunflower lecithin, carnauba wax. Xylitol, gum base, vegetable glycerin, natural anise & licorice flavors, gum arabic, sunflower lecithin, carnauba wax Xylitol, gum base, vegetable glycerin, natural anise & licorice flavors, gum arabic, sunflower lecithin, carnauba wax

2) Carbohydrate drink

(1) Name: NoNPO

(2) Ingredient: water, dextrin, sucrose, tomatine, sucralose, maltodextrin

**6. Study participants**

1) Inclusion criteria

(1) ASA class I-III adult patient (age 18 and under 70)

(2) Patients undergoing the laparoscopic gynecological surgery

2) Exclusion Criteria

(1) Pediatric (under 18)

(2) Old age (over 70)

(3) Pregnant and nursing mothers

(4) Patients with unstable vital signs (emergent conditions)

(5) Patients who are at high risk of aspiration: persons with a BMI of 30 or more, gastroesophageal reflux, achalasia, enteropathy, diabetes mellitus, and with a history of gastrointestinal tumor

(6) current medication affecting gastrointestinal motility

(7) uncontrolled liver or kidney disease

(8) Emergency surgery

(9) Patients who do not agree to participate in the study

3) Protection measures when recruiting vulnerable participants

All study subjects will obtain informed consent 1 day in advance and will spend at least 30 minutes sufficient time for explanations. In the case of subjects with reduced understanding and judgment, additional protective measures such as obtaining additional consent from the guardian (or representative) will be taken.

**7. Sample size calculation**

1) Primary hypothesis: The anxiety level of the group chewing gum while taking oral carbohydrate drinks during pre-anesthetic fasting will be lower than that of the group taking oral carbohydrate drinks.

2) Based on the results of the previous study, the mean of anxiety measured by VAS immediately before surgery when taking carbohydrate drinks was 34.83, and the standard deviation was 14.8. In addition, in the case of women, it was reported that the average value of anxiety before surgery was about 15% higher. Based on this, the VAS of preoperative anxiety in the female patient group immediately before surgery was corrected to 39.31 ± 17.04. Forty-seven patients in each group are needed to find the difference in the anxiety of 25% between the two groups, with 5% of alpha and power of 80%. Estimating a dropout rate of 10%, a total of 104 patients are needed, 52 in each group.

**8. Study period**

1 year from the clinical trial approval date

**9. Methodology**

1) Time flow

1. The patient is assigned to either the experimental group or control group on a random basis on the eve of the operation. All patients scheduled for gynecologic surgery were instructed to fast overnight before surgery, and oral carbohydrate drink (NO NPO^Ⓡ^: Daesang, Seoul, South Korea) loading was used routinely according to the protocol of the department of gynecology
2. The patients in the control group (CHD group) are requested to follow the aforementioned fasting guideline. The participants were instructed to consume at least 600 mL to 800 mL of carbohydrate drinks up to 3 hours before induction of anesthesia.
3. The patients in the test group (CHD with gum group) follows the fasting protocols of our gynecologic strategy and were encouraged to chew gum freely with oral carbohydrate loading during fasting time.
4. There is no intervention other than chewing gum during fast times between the two groups of patients.
5. Gum will be removed right before being transported to the operating room before transfer. The nurse in charge of the operating room double-checks whether or not gum has been removed during the patient identification process in the preoperative holding area.
6. The severity of anxiety would be examined at the preoperative holding area immediately before surgery.
7. In the operating theater, all participants are provided with supplemental oxygen. During pre-oxygenation, a gastric ultrasound exam is performed by YJ B.
8. The independent anesthesiologist will induce general anesthesia according to the standardized protocol. Propofol and remifentanil are used using a TCI machine with the aim of a Bispectral Index of 40-60. Blood pressure and heart rate were maintained within 10% of baseline. The blinded anesthesiologist will rate the oral secretion during tracheal intubation.
9. Gastric juice is naturally drained via an esophageal temperature probe (ST probe) during surgery and analyzed with an acidity analyzer.
10. In the course of surgery and maintenance of anesthesia, the subsequent process proceeds without special intervention. Total anesthesia time, operation time, drugs used during anesthesia, and vital signs are collected through medical records. (Attachment of case reporting form)
11. Afterward, the treatment and recovery of patients in the ward will be proceeded according to the general guidelines of the department of obstetrics and gynecology based on the ERAS protocol.

- **standardized anesthesia protocol**

Standard ASA monitoring including noninvasive blood pressure, EKG, pulse oximetry, and bispectral index (BIS) monitoring will be applied. After denitrogenation with 80 % oxygen, general anesthesia will be induced with propofol and remifentanil using target-controlled infusion (Orchestra® Base Primea; Fresenius Kabi, Brezins, France), and intravenous rocuronium 0.8 mgkg^-1^. Another investigator who is not aware of group allocation will perform intubations and evaluate the degree of oral secretion during intubation. After tracheal intubation via video stylet, the ventilator was set with a tidal volume of 8 mLkg^-1^of ideal body weight and FiO_2_ 40 %. The respiratory rate and I: E ratio were adjusted to maintain inspiratory peak pressure of less than 30 cmH_2_O and normocapnia. During the whole surgery, propofol and remifentanil effect site concentrations will be adjusted to achieve BIS values of 40 to 50 and to maintain mean blood pressure and heart rate within 20 % of pre-induction values.

- **Postoperative management protocol**

Postoperative analgesia is standardized for all patients. If patients presented with breakthrough pain (NRS ≥ 4/10), IV ibuprofen 400 mg will be administered. If this proved ineffective after 30 minutes IV pethidine 50 mg will be administered. Postoperative nausea and vomiting are treated with intravenous metoclopramide 10 mg and ramosetron 0.3 mg. All patients resume diet and ambulation as soon as possible after full recovery from anesthesia unless gastrointestinal symptoms are noted. Hospital discharge is determined by the surgery team.

2) Randomization and blinding

The patient is assigned to either the experimental group or control group on a random basis on the eve of the operation. There is no intervention other than chewing gum during fast times between the two groups of patients, and subsequent anesthesia and pre-anesthesia examinations will be conducted according to a standardized protocol. The randomization list was generated by a random permuted block design with a block size of two. The allocation information is sealed in an opaque envelope and piled up in a cabinet. Gynecologist open the sealed envelope, in turn, to confirm the assignment information and distributed or not distributed chewing gum, accordingly. Randomization information is managed so that the list is not disclosed to the evaluator and only primary investigators can know. After entering the operating room, both the control group and the test group proceed according to the same study protocol, and both gastric volume measurements and acidity records are recorded by the YJB. Anxiety levels prior to surgery and uncomfortable symptoms related to fasting will be recorded by an anesthesiologist who is not participating in the study based on patient self-report. Postoperative gastrointestinal motility recovery and postoperative recovery questionnaires were completed by the patient under the guidance of an independent maternity ward nurse and attending physician.

**10. Outcomes and assessment**

1) Patient characteristics: Age, sex, height, weight, BMI, ASA class, and other comorbidities,

smoking, alcohol

2) Gastric fluid volume & pH

After taking the right lateral decubitus position, the diameter of the antrum is measured by abdominal ultrasonography.

The gastric fluid volume is calculated using the following formula.

* volume (ml) = 27 + 14.6 9 * antral area (cm²) – 1.289 * age (year)

After anesthesia induction is completed, gastric fluid is collected through an esophageal probe ad acidity is measured using a pH meter.

3) Pre-operative anxiety

(1) Anxiety with VAS

(2) Anxiety with APAIS score


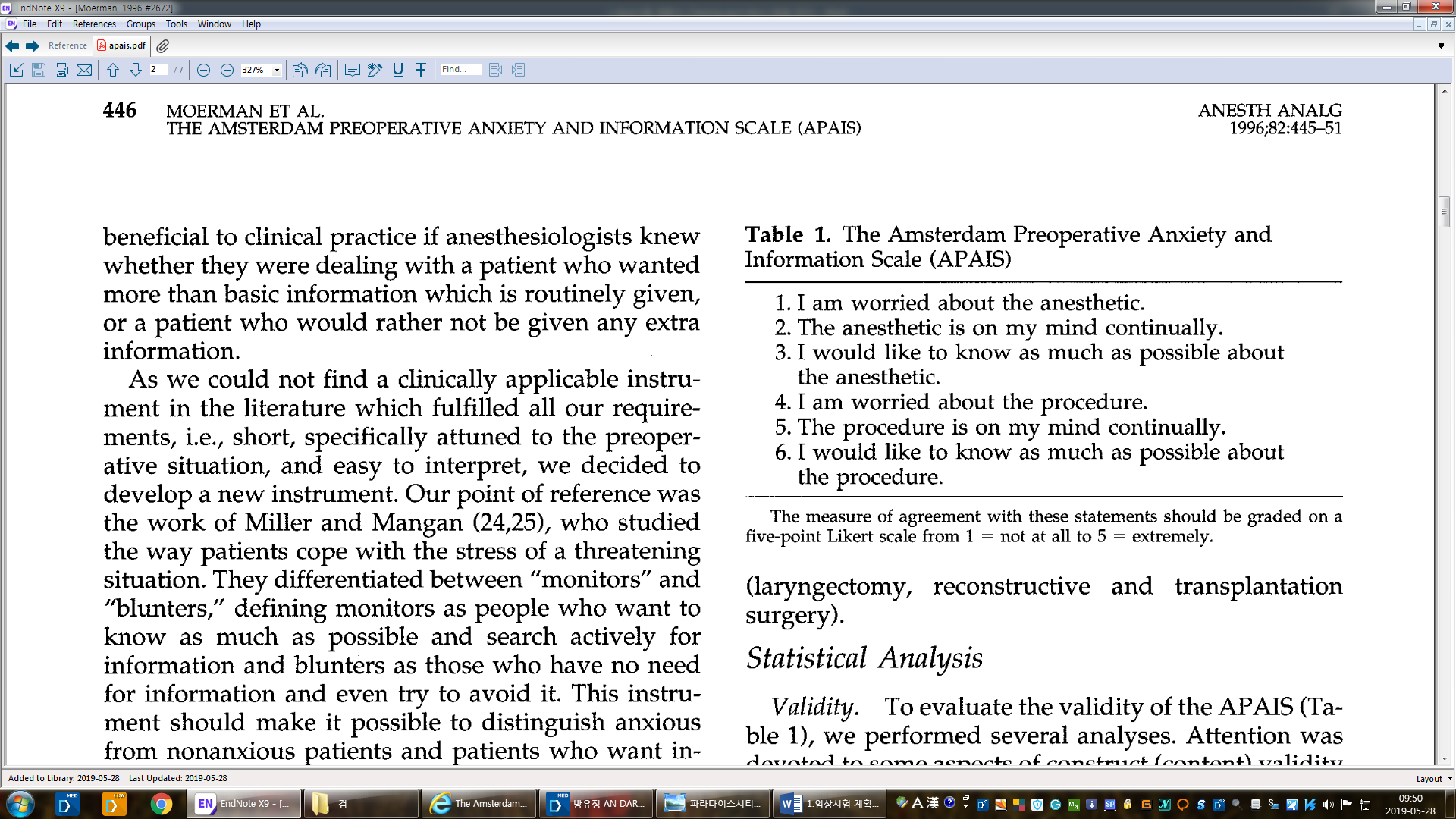


The APAIS score was developed to measure patients' anxiety before surgery, and its effectiveness has been verified in several studies. The APAIS score is calculated by summing the scores from 1 to 5 for 6 questions. a

(3) Symptoms related to anxiety

Whether chewing gum during fasting helped to endure the fasting time, difficulty concentrating, and anxiety-related symptoms such as hunger, thirst, dry mouth, fatigue, headache, nausea, etc. were surveyed with a score between 0 and 10 for each item. do. (Questionnaire Attachment 1)

4) Recovery or bowel function

Post-operative nausea & vomiting

Flatus expulsion, defecation time in postoperative time, time of resume feeding

Intra-hospital bowel complication (ileus, dyspepsia, abdominal pain)

5) QoR15

QoR 15 is calculated by the total sum of the patient's postoperative recovery by asking questions on a scale from 0 to 10 for each item. The higher the score, the higher the quality of recovery.

6) Other Observation Items

(1) The amount of oral secretion at the time of intubation (none/ mild/ moderate/ severe)

(2) Total dose of the anesthetic drug used during anesthesia, amount of fluid

(3) Total anesthesia time and operation time

(4) Total amount of opioids used in the intrahospital period

7) Timeline

**11. Anticipated side effects and precautions for use**

In this study, patient information will be investigated the day before anesthesia, because patients with a high risk of pulmonary aspiration (obesity, gastrointestinal disease, DM, and hepatic renal disease) will be excluded from this study in advance. Thus, the risk of pulmonary aspiration does not increase. However, as a way to cope with the possibility of aspiration, in this study, an additional anesthesiologist will assist by using the Sellick maneuver and will be able to cope with an emergency event. For assuring patient safety, we will examine the gastric fluid volume, gastric acidity, and the amount of oral secretion at tracheal intubation. A gastric ultrasound scan will be performed immediately before induction of general anesthesia. If the participants had confirmed not having empty stomachs, we decided to delay the operation or perform general anesthesia using the rapid sequence induction method. Gastric acidity was checked with the aspirate of an orogastric thermometer.

**12. Suspension/Dropout Criteria**

1) In case of a hemodynamically unstable state before and after anesthesia

2) When a previously undiagnosed patient has an allergic reaction to an anesthetic agent

3) Subject's voluntary withdrawal of consent

4) Failure to follow the instructions of the research doctor

5) Occurrence of a serious disease unrelated to study participation

6) In the case that Your doctor has decided that research is not the best option for participants.

**13. Outcomes and statistical analysis**

1) Primary outcome: Anxiety with APAIS score, Symptom related to anxiety

2) Secondary outcome: Flatus expulsion or defecation time in postoperative time, time of resume feeding, Intra hospital bowel complication, gastric fluid volume & pH, oral secretion at intubation time, QoR 15 score

3) Analysis

All continuous data are compared with Student’s t-test or Wilcoxon’s rank sum test according to normality. All categorical variables are analyzed using the chi-square test, Fisher’s exact test. The descriptive variables will be presented as mean ± standard deviation or median with an interquartile range. The categorical data are summarized as the number (%). The difference in APAIS, the primary outcome will be compared using student’s t-test. The multiple comparisons for sub-scores of APAIS would be adjusted with Bonferroni correction.

The association between QoR-15K and APAIS scores measured in the preoperative holding area will be analyzed using linear regression model.

**14. Safety evaluation criteria, evaluation methods, and reporting methods including side effects**

This study is a prospective study with an intervention called chewing gum during the fasting period. Pulmonary aspiration of gastric contents is not predicted because there is no difference in side effects compared to the classical fasting anesthesia method according to the results of previous studies. However, since there is a risk of airway obstruction due to chewing gum in the mouth during anesthesia induction, it will be evaluated whether the chewing gum has been removed three times in the pre-anesthetic stage. Even during the anesthesia process, the patient's airway will be observed with a video assist device, and tracheal intubation will be performed after evaluating the airway obstruction of the chewing gum. In all patient groups participating in this clinical trial, vital signs such as blood pressure, pulse rate, oxygen saturation, and respiratory rate will be thoroughly monitored and evaluated immediately after entering the operating room, during surgery, and until leaving the recovery room. After this, the medical staff will visit and investigate side effects up to 72 hours after surgery. During this study, the clinical trial director and the person in charge will make every effort to ensure the safety of the subjects, and in the event of a serious adverse reaction, the study will be stopped and prompt and appropriate measures will be taken to minimize the adverse reaction. In addition, serious adverse reactions will be reported to the IRB according to the institutional guidelines.

**15. Subject Consent Form**

Attach a separate form

**16. Code of Victim Compensation**

1) Compensation standard:

If there is a compensation amount or measures agreed upon in advance between the parties for adverse reactions of intervention (intervention) according to clinical research, compensation is made according to the relevant standards. In other cases, compensation shall be made according to the compensation method agreed between the parties after comprehensively considering the degree, nature, duration, and similar cases of physical damage. If the agreement in the preceding paragraph is not reached between the parties, compensation shall be made according to the judgment of the court and the conclusive content of the decision corresponding thereto.

2) Compensation source:

Use the in-hospital compensation standard. (If emergency treatment is required due to damage related to this clinical trial, Samsung Medical Center will bear the cost of emergency treatment for the first 24 hours at Samsung Medical Center. In the case of side effects, known treatment methods will be treated.)

**17. Case report form**

Attach a separate form

**18. Criteria for treatment and treatment of subjects after clinical trials**

We anticipate that no special care or treatment will be required.

**19. Matters necessary to conduct other clinical trials safely and scientifically**

In the previous study, there was 1 case where chewing gum was found while under anesthesia, so chewing gum should be avoided. The chewing gum is removed right before being transferred to the operating room. The first check is made by the ward nurse, and the second check is made by the operating room nurse in the waiting room of the operating room. Before administering an anesthetic agent, the anesthetist in charge checks the removal of the gum three times to prevent gum aspiration accidents. Patients participating in this clinical trial will be continuously monitored and evaluated for vital signs such as blood pressure, pulse, oxygen saturation, and respiratory rate from before induction of anesthesia to the point of leaving the recovery room. In addition, if unexpected stability problems are found in this study, the clinical trial will be immediately stopped and reported according to the serious adverse reaction report form.

**20. Measures for the safety and protection of participants**

The data obtained in this study will not be used for any purpose other than research and will be coded and recorded in numbers to prevent exposure of patients' personal information on the case record sheet, and records that can identify the subject will be kept confidential
